# Law-breaking, fairness, and generalized trust: The mediating role of trust in institutions

**DOI:** 10.1371/journal.pone.0220160

**Published:** 2019-08-07

**Authors:** Sergio Lo Iacono

**Affiliations:** Department of Political and Social Sciences, European University Institute, Florence, Italy; London School of Economics, UNITED KINGDOM

## Abstract

Institution-centered accounts of generalized trust rely on the idea that law-breaking and state’s unfairness lower individuals’ propensity to trust fellow citizens because of a weaker confidence in the state. Despite the theoretical relevance attributed to this mediation mechanism, no empirical analysis in the literature has focused on examining its correlational validity. Using data from the European Social Survey (2010), the Quality of Government EU Regional data, and EUROSTAT, this paper assesses the intervening role of institutional trust on the relationship between crime rates, state’s fairness, and generalized trust. Results from a Multilevel SEM (MSEM) mediation analysis indicate that trust in institutions strongly mediates the relationship between violent crimes (i.e. homicide) and generalized trust but not the one between property crimes (i.e. vehicle thefts and robberies) and generalized trust. On the other hand, indicators of fairness (i.e. impartiality and corruption) are all mediated by institutional trust, though impartiality maintains a significant direct effect. Overall, findings support the institutional approach, confirming that the negative relationship between ineffective and unfair institutions and generalized trust passes mostly through people’s lost faith in the state.

## Introduction

Most of our daily social interactions involve anonymous others of which we know nothing or very little about. Despite this uncertainty, cooperation is not rare. In fact, contemporary societies tend to deeply rely on positive impersonal interactions among strangers who believe that their positive expectations about the behaviors of other actors will be reciprocated [1]. This propensity to trust people we do not know, that is to place generalized or social trust, has been argued to be a central feature of a prosperous society [2–4].

Empirical research has shown that when a society is not regulated in a way that fosters the good of all its members and the norms of communal living are frequently broken, social trust declines. For instance, studies looking at the impact of material deprivation, unemployment, crime and poverty rates on trust indicate that in areas where this social upheaval is more prominent, people are less likely to believe in the trustworthiness of their unknown fellow citizens, regardless of their personal condition [5–10].

Noticeably, the institution-led perspective claims that when institutions are universally oriented, and act as a fair and effective enforcer of the law, people will be more prone to think that the state will successfully intervene to decrease opportunism and arbitrate disputes. In turn, this will encourage the belief that trusting strangers involves fewer risks [11–15]. According to this line of thought, when in a certain area there is a lack of goods that entails a scarce ability of the state to fulfil its functions, individuals’ lower propensity to trust fellow citizens should be due to a weaker confidence in the state.

Though this argument strongly relies on the intervening function of institutional trust, virtually no empirical analysis has directly tested the validity of the mediation effect. Indeed, while several studies show a direct impact of institutional quality on social trust, they do not assess if and to what extent institutional trust is intervening on such relationship. As a matter of fact, there is a major lack of research in this respect, as we do not have evidence of significant *correlational relationships* confirming (or disconfirming) the mediation argument. This has left unclear whether it is plausible to assume that the association between poor macro-level conditions and individuals’ social trust passes through a common factor, namely institutional trust. That is, do worse social conditions lead us to distrust other citizens because of our lost faith in the state? Or is this more likely due to the specific challenges posed by such social disruptions?

This is the first exploratory study investigating the mediating role of institutional trust on the relationship between crime rates, state’s unfairness, and generalized trust. Using data from the European Social Survey (ESS– 2010) in combination with regional information (NUTS II level) from EUROSTAT and the Quality of Government dataset [16], we employ Multilevel Structural Equation Modelling (MSEM–fitted via Mplus 8.3) to address this gap in the literature. In particular, moving beyond traditional methods to assess mediation [17, 18], we follow Preacher and colleagues to properly test *multilevel mediation* and use MSEM to separate the between and within component of the intervening variable, avoiding bias in the calculation of the indirect effect [19]. Throughout the paper we refer to direct and indirect effects to discuss mediation in accordance with the predominant language used in the literature. However, no claim of causality is made. Conversely, this analysis aims at testing the correlational validity of the mediation argument. In doing so, we examine a traditional institution-centered argument, and assess how the deficiency of state’s protection of citizens (i.e. violent and property crimes) and institutions’ fairness (i.e. institutions’ impartiality and corruption) correlates with individuals’ generalized trust, and if and to what extent this association is attributable to institutional trust.

## Law-breaking and institutional fairness

Institutional accounts of social trust have been developed over the last decades as an alternative to the society-centered approaches [3, 20], which emphasize the relevance of social connections (e.g. voluntary associations) in the formation of trust among fellow citizens. On the other hand, the institution-centered perspective asserts that “the state can favor the development of social trust by sanctioning those who do not honor trust placed in them. If we know that any non-compliance with an agreement will be sanctioned by the state, we will have greater expectations about other people’s compliance” [21]. “Nevertheless, in real life, states are not perfect enforcers of agreements” [22], and they are able to punish defectors only in some instances. Thus, people place trust in their fellow citizens only when there are good chances that the state will intervene. Yet, assessing such probabilities is a rather complex process, which requires citizens to rely on a series of factors, such as past institutional performances, values and criteria followed, and so on.

One of the aspects most widely emphasized across the literature as pivotal in the promotion of trust is the capacity of the state to act as an effective enforcer: if institutions are unable to practically prevent law-breaking, individuals’ evaluation of state’s reliability should tend to be negative and therefore social trust would not be fostered [12, 14]. For instance, Freitag and Bulhmann, argue that “if there is reason to suspect that the rule of law in a given country is weak, such that legal organs like the judicial system or law enforcement are unable to ensure secure contracting […], mistrust between individuals is more likely to develop” [11]. Likewise, Robbins suggests that as long as institutional incentives allow people to feel safe in their interactions with anonymous others, then social trust will be encouraged [13].

Along similar lines, scholars have emphasized the relevance of state’s fairness in promoting trust towards fellow citizens [14]. This position rests on the idea that if institutions do not treat everyone according to the same set of principles and do not allow equal opportunities, people will believe that the state will side with one actor over the others, favoring the development of treacherous attitudes and behaviors [13, 15]. Using subjective-based measures of institutional quality, past empirical research has corroborated such claims by showing that in areas connoted by more effective enforcement of private agreements, protection of legal rights, and lower levels of corruption, trust in our unknown fellow citizens is more likely to emerge [13, 15, 21, 23].

However, while these inquiries thoroughly investigate the direct impact of institutions on social trust, they neglect the intervening role of trust in institutions on the relationship, leaving untested the mediation mechanism. A partial exception seems to be represented by You’s work “Corruption and inequality as correlates of social trust”[24], where using data from the World Value Survey (1995–1997 and 2000–2001) and the European Value Survey (1999–2000), he runs several hierarchical models to survey how higher levels of corruption at the country-level influence individuals’ political and social trust. Other studies that have taken into account some of the same concepts considered in You’s analysis appear to be in line with his results while evaluating separate parts of the mediation argument. In particular, they show that (1) institutional quality and social trust are positively and significantly correlated in cross-sectional and panel analyses [11, 15, 22, 25, 26]; (2) macro-institutional factors have a relevant impact on trust in political institutions [27–29]; (3) political and social trust are positively and strongly associated [30–33]. If taken together these studies are encouraging and provide probationary evidence for the mediation mechanism, even though they do not test directly the meditation effect [34]. In this sense, despite the large body of research on institutional quality and trust, the literature still lacks of an empirical analysis evaluating the significance and the scope of the intervening role of institutional trust.

Building upon previous contributions, this article addresses this gap, and assesses the correlational validity of the mediation mechanism by testing if and to what extent reported association between crime rates, state’s fairness and social trust are intermediated by trust in institutions. Crime rates (e.g. homicide, thefts or robberies) are often taken as an indicator of the state’s inability to assure citizens’ safety, and their negative correlation with social trust is well-documented in the literature [6, 9, 35]. According to the institutional framework, this detrimental impact should be attributed to a general mistrust in the institutions. That is, living in an area with more homicides or robberies would lead us to believe that the state is generally unable to carry out its job, creating a lower propensity to rely on institutions. As a result, we will be more likely to feel unprotected in our exchanges with our unknown fellow citizens, and distrusting strangers would appear the safest option to reduce hazards [13]. Empirically, if this interpretation is correct, we should find some confirmation to the following hypothesis: (H1) *individuals living in regions with higher violent or property crime rates are less likely to trust institutions*, *and less likely to place generalized trust*.

In a similar vein, several analyses showed the existence of a direct impact of higher levels of unfairness and inequality on social trust [11, 26, 36]. Thus, if the mediation mechanism is effectively at work, when institutions are strongly corrupted and tend to systematically side with one party over the other, individuals should be more skeptical of the state’s capacity to act as a fair third-party enforcer in arbitrating disputes. Ultimately, this would facilitate the emergence of suspicious and distrusting attitudes towards fellow citizens, since people cannot know whether institutions will correctly intervene to protect them in case of need [13]. In other words, we should expect that (H2) *individuals living in regions with higher levels of corruption or lower impartiality are less likely to trust institutions*, *and less likely to place generalized trust*.

## Material and methods

The analysis is conducted using the Quality of Government EU regional data (QoG), official statistics from the EUROSTAT, and the 5^th^ round of the European Social Survey (ESS—2010).

The ESS is a cross-national survey carried out in Europe each two years from 2002 collecting data on people’s demographics, opinions and attitudes on a variety of issues (e.g. justice, confidence in public institutions etc.). The EUROSTAT provides information on regional demographics, economic indicators (e.g. GDP and unemployment) as well as integrated crime statistics across NUTS II regions. Finally, the QoG (2010) offers data on the quality of institutions at the regional level on the basis of a survey involving 34,000 participants across 147 NUTS II regions [16]. The NUTS classification (Nomenclature of territorial units for statistics) is a hierarchical system composed by three levels (NUTS I, NUTS II, NUTS III) that subdivides each country into a certain number of NUTS I regions, each of which is in turn subdivided into a number of NUTS II regions, and so on. We excluded NUTS II regions that correspond to an entire country (e.g. Lithuania and Estonia) or present inconsistencies with the ESS dataset due to modification of NUTS coding (e.g. Finland and Croatia) after the implementation in 2012 of the ‘NUTS nomenclature 2010’, which changed the subdivision of the territory of the European Union at the regional level.

Though these datasets have been running for different periods of time, they all provide information for the year 2010, which has been therefore selected for this study. Notice, however, that regional data for median age, population growth, percentage of migrants from inside and outside the EU, and percentage of the active population holding a managerial or professional position, are taken from the Census 2011 as provided by the EUROSTAT. Since these figures rely on the 2011 Census, they are more accurate than estimates available for the year 2010. We restrict our sample to respondents aged over 17 for regions where information on variables of interest are available (see Table 1).

**Table 1:** Sample size, countries and NUTS II regions.

Country	Number of Individuals	Number of NUTS II regions	Average number of individuals per NUTS II
Belgium*	1,639	11	149
Bulgaria	2,393	6	398.8
Czech Republic	2,320	8	290
Denmark	1,506	5	301.2
Spain	1,831	17	107.7
France	1,679	21	80
Hungary*	1,531	7	218.7
Netherlands*	1,789	12	149.1
Poland	1,656	16	103.5
Portugal	2,091	5	418.2
Sweden*	1,430	8	178.8
Slovenia*	1,354	2	677
Slovakia	1,823	4	455.8
(TOTAL)			
13	23,042	122	188.9

*not available in the QoG 2010 dataset at the NUTS II level for the impartiality and corruption pillars of the European Quality of Government Index.

Preliminary exploration of data revealed a significant Intra-Class Correlation for NUTS II regions equal to 17 per cent for the dependent variable ‘Generalized Trust’, indicating that analyzing regional level differences represents an extremely important line of inquiry (though often overlooked). Also, it seems relevant to point out that focusing on regional level data offers a number of advantages over national level data by granting a larger sample size and higher statistical power as well as the possibility to capture heterogeneity within countries (a point particularly significant for states connoted by substantial internal differences, such as France or Spain). NUTS II regions have been preferred to NUTS III regions for at least two reasons: (1) NUTS II regions tend to correspond to areas where regional policies are applied, potentially establishing a link between the lack of a specific good and individuals’ view on the state. (2) NUTS II regional information are richer (i.e. more variables are accessible for this level of analysis) and available for more countries (creating more variability).

In order to take into account the lack of independence among observations and analyze how macro-factors at the regional level affect individuals’ propensity to trust, we employ a recursive multilevel structural equation model where individuals (level 1) are nested within NUTS II regions (level 2). Given the multilevel design of the mediation model, we follow Preacher and colleagues and calculate the effect of the intervening variable by separating its between and within component. This allows us to avoid conflation and obtain an unbiased estimate of the between indirect effect [19].

The dependent variable, social trust, is measured by using an 11-points scale based on the standard question: ‘Generally speaking, would you say that most people can be trusted, or that you can’t be too careful in dealing with people?’. Institutional trust, the mediation variable, is obtained by combining respondents’ reported trust in the parliament, legal system and parties (see Table 2 and Supporting Information for more details). The homicide rate is estimated by dividing the number of homicides reported to the police by the total number of residents in the region. The result is then multiplied by 100,000. The same process is followed for property crimes, which include robberies and vehicle thefts. Our operationalization of state’s fairness relies on two dimensions of the European Quality of Government Index, namely the impartiality and corruption pillars. These are based on two batteries of questions measuring the perceived degree of impartiality and corruption in the respondents’ area regarding law enforcement, public health, public education and so on [16] (see https://qog.pol.gu.se/data/datadownloads/qog-eqi-data for more details). High values of corruption indicate a lack of corruption, whereas high values of impartiality indicate a strong impartiality of institutions.

**Table 2:** Demographics and operationalization of main concepts.

Variables’ description	Mean	S.D.	Range	n individuals (NUTS II)
Generalized Trust: 0 = Can’t be too careful; 10 = Most people can be trusted	4.69	2.48	0–10	23,042 (122)
Trust in Parties: 0 = No trust at all; 10 = Complete trust	3.18	2.35	0–10	22,602 (122)
Trust in the Legal System: 0 = No trust at all; 10 = Complete trust	4.47	2.65	0–10	22,461 (122)
Trust in the Parliament: 0 = No trust at all; 10 = Complete trust	3.95	2.54	0–10	22,505 (122)
Age	49.82	17.96	18–96	23,008 (122)
Male	0.46	0.50	0–1	23,037 (122)
Unemployment (not being employed in the last 7 days)	0.08	0.28	0–1	23,042 (122)
Member of a discriminated group	0.06	0.24	0–1	22,914 (122)
Homicides per 100,000 residents: Number of homicides reported to police/ Resident Population * 100,000	1.21	0.60	0.28–3.10	21,253 (110)
Property crimes per 100,000 residents: Number of robberies and vehicle thefts reported to police/ Resident Population * 100,000	249.55	221.99	23.27–1,088.17	23,042 (122)
European Quality of Government—Corruption Pillar	-0.38	1.19	-2.26–2.22	15,299 (82)
European Quality of Government—Impartiality Pillar	-0.45	1.10	-2.58–2.04	15,299 (82)
Long-term Unemployment: Number of registered unemployed for 12 months or more/Number of active people	4.27	2.77	0.87–12.33	23,002 (121)
Population growth–Crude rate of natural change	0.65	3.35	-11-9.6	23,042 (122)
GDP at current market price: Euro per inhabitant in % of the European average	84.10	52.33	12–251	23,042 (122)
Percentage of people from an Extra-EU country	0.03	0.03	0.001–0.13	23,042 (122)
Percentage of people from another EU country	0.02	0.02	0.001–0.20	23,042 (122)
Percentage of active population holding an upper secondary degree or above	0.74	0.17	0.26–0.97	23,042 (122)
Percentage of economically active people holding a managerial or professional position	0.22	0.06	0.13–0.46	21,403 (111)
Median age of population	40.28	2.19	34.8–46.6	23,042 (122)

Sources: ESS, EUROSTAT, QoG regional data.

To address possible sources of bias and adequately separate the role of regional factors from individual ones, on the basis of previous literature, we select and control for covariates that might affect the dependent, independent and mediation variables. In this sense, we include measures of GDP per capita, population growth, median age, percentage of migrants from inside and outside the EU, percentage of the active population (i.e. people between 25–64 years old) with a secondary degree or above, percentage of economically active population (i.e. people between 15–64 years old) holding a managerial or professional position, long-term unemployment, and regions’ size at the NUTS II level [15, 37–40]. Likewise, at the individual level, we apply controls for gender, education, age, attitudes towards migrants, self-perception of health status, fear of crime, employment, being member of a group discriminated against in the country (e.g. for racial or religious reasons) and social connections [3, 31, 32]. Education has the following values: 0 “lower secondary or less” 1 “upper secondary” 2 “advanced vocational, sub-degree” 3 “Bachelor, Master or higher”. Perceived health status is a 5-points scale based on the following question: “How is your health in general?”, 1 indicates “very good” and 5 “very bad”. Pro-migrants views is an index combining together three different questions (see Supporting Information), namely “would you say it is generally bad or good for [country]'s economy that people come to live here from other countries?”, “would you say that [country]'s cultural life is generally undermined or enriched by people coming to live here from other countries?”, and “is [country] made a worse or a better place to live by people coming to live here from other countries?”. All these questions rely on a 11-points scale. Fear of crime is a 4-points scale based on the question “How safe do you—or would you—feel walking alone in this area after dark?” where a value of 1 means “very safe” and a value of 4 means “very unsafe”. Finally, the variable social connections indicates how often respondents socially meet with friends, relatives or colleagues, and it ranges between 1 “never” and 7 “every day”.

## Results

Table 3 shows results from a two-level structural equation model on generalized trust with violent and property crimes as predictors and institutional trust as a mediating variable. In all models we apply listwise deletion of missing values on variables of interest.

**Table 3:** MSEM testing the mediation effect of institutional trust on the relationship between crime and social trust.

	Model 1 No Mediation	Model 2 With Mediation	Model 3 No Mediation	Model 4 With Mediation
Individual Level				
*SOCIAL TRUST ON*				
Age	0.005** (0.002)	0.002 (0.002)	0.036** (0.012)	0.002 (0.001)
Male	-0.085* (0.040)	-0.063 (0.040)	-0.020* (0.008)	-0.075* (0.038)
Education	0.198*** (0.020)	0.179*** (0.019)	0.092*** (0.008)	0.167*** (0.018)
Unemployment	-0.174*** (0.048)	-0.136** (0.048)	-0.022*** (0.006)	-0.136** (0.047)
Perceived Health Status	-0.233*** (0.033)	-0.103*** (0.026)	-0.095*** (0.013)	-0.161*** (0.031)
Social Connections	0.031 (0.018)	0.032 (0.019)	0.021 (0.012)	0.031 (0.018)
Discriminated Group	-0.297** (0.086)	-0.172* (0.080)	-0.032*** (0.008)	-0.179* (0.073)
Pro-migrants	0.257*** (0.020)	0.182*** (0.021)	0.217*** (0.014)	0.184*** (0.020)
Fear of Crime	-0.233*** (0.033)	-0.261*** (0.030)	-0.100*** (0.010)	-0.267*** (0.029)
Institutional Trust	-	0.331*** (0.022)	-	0.339*** (0.021)
NUTS II Level				
*INSTITUTIONAL TRUST ON*				
% Migrants from outside EU	-	-7.072 (4.288)	-	-11.891* (4.836)
% Migrants from inside EU	-	5.450 (5.018)	-	10.257 (6.333)
% Managers or Professionals	-	-3.136** (1.198)	-	-3.437** (1.259)
% Upper degree or above	-	0.009* (0.004)	-	0.005 (0.005)
GDP	-	0.020*** (0.002)	-	0.022*** (0.003)
Population Growth	-	-0.155*** (0.030)	-	-0.103** (0.036)
Age Median	-	-0.150*** (0.035)	-	-0.132*** (0.040)
Area in Km2	-	0.000 (0.002)	-	0.001 (0.002)
Long-term Unemployment	-	-0.005 (0.032)	-	-0.036 (0.036)
**Intentional Homicides**	-	-0.361** **(0.116)**	-	-
**Property Crimes**	-	-	-	**0.001 (0.001)**
*SOCIAL TRUST ON*				
% Migrants from outside EU	0.311 (2.849)	6.988 (3.785)	-0.093 (0.081)	6.758 (3.818)
% Migrants from inside EU	7.784 (4.737)	2.237 (4.266)	0.164 (0.086)	1.299 (4.506)
% Managers or Professionals	-0.585 (1.204)	2.416* (1.005)	-0.012 (0.058)	2.789** (0.909)
% Upper degree or above	0.005 (0.004)	-0.003 (0.004)	-0.024 (0.064)	-0.005 (0.004)
GDP	0.016*** (0.002)	-0.004 (0.196)	0.747*** (0.075)	-0.003 (0.003)
Population Growth	-0.194*** (0.028)	-0.033 (0.028)	-0.370*** (0.071)	-0.022 (0.024)
Age Median	-0.184*** (0.041)	-0.034 (0.036)	-0.314*** (0.076)	-0.021 (0.032)
Area in Km2	0.003 (0.003)	0.003 (0.002)	0.026 (0.054)	0.001 (0.002)
Long-term Unemployment	-0.032 (0.035)	-0.022 (0.023)	-0.116 (0.077)	-0.015 (0.023)
**Intentional Homicides**	-0.383** **(0.121)**	**-0.025 (0.088)**	-	-
**Property Crimes**	-	-	-0.156* **(0.073)**	-0.001** **(0.0004)**
**Institutional Trust**	-	0.979*** **(0.119)**	-	0.905*** **(0.098)**
**Intentional Homicides Indirect Effect**	-	-0.354** **(0.114)**	-	-
**% of Total Effect**	-	**93%**	-	-
**Property Crimes Indirect Effect**	-	-	-	**-0.0002 (0.0004)**
**% of Total Effect**	-	-	-	**17%**
n individuals (NUTS II)	17,087 (99)	16,534 (99)	18,734 (110)	18,148 (110)
RMSEA	0.030	0.021	0.031	0.021
R2 Between	0.824	0.940	0.787	0.945
R2 Within	0.097	0.156	0.099	0.160

Note. Unstandardized Coefficients. Standard errors in parentheses. MLR.

* *p* ≤ 0.05,

** *p* ≤ 0.01,

*** *p* ≤ 0.001.

Models 2 and 4 account also for mediation of institutional trust at the individual level. Sources: ESS, EUROSTAT, QoG regional data.

At the individual level, the analysis points out the relevance of usual predictors of social trust: age, gender, perceived health status, and being part of a discriminated group are all significantly correlated with generalized trust in the expected directions. Furthermore, Model 1 shows that being unemployed or worried about crime have a negative influence on individuals’ propensity to rely on fellow citizens, while higher educational levels or having pro-migrants views are positively associated with social trust. Only social connections is insignificant, most likely because of the wide set of controls applied. At the NUTS II level, Model 1 illustrates that regions with an older population (i.e. median age) and a higher population growth display a lower level of trust. Inversely, GDP per capita has a positive coefficient in the equation, indicating that living in areas with higher overall levels of wealth is beneficial to trust our unknown fellow citizens (p < 0.001). Other covariates, such as percentage of migrants from inside or outside the EU, have an insignificant role in the equation–in accordance with Hooghe et al.’s analysis [39].

As regards H1, the model points out a clear negative relationship between intentional homicides and social trust (p < 0.01), showing that the emergence of trust in fellow citizens is strongly discouraged in regions with more intentional homicides. This is in line with prior research. To assess if this relationship can be attributed to a lower confidence in institutions, we allow for mediation in Model 2 by adding relevant paths in MSEM and separating the cluster-level component of institutional trust from the individual-level one [19]. As it can be noticed, the introduction of institutional trust has a strong impact on the equation, displaying a significant correlation with social trust as well as other covariates. Model 2 indicates that people who tend to believe that they can rely on the legal system, parliament, and parties of the country are very prone to place positive expectations towards strangers. More importantly, including trust in institutions absorbs almost entirely the effect of intentional homicides on social trust, whose impact passes through trust in institutions for about 93% of the total effect (p < 0.01). This intervening role of institutional trust is robust to different specifications (e.g. including or excluding other covariates in the equation does not change the significance of the mediation), and it is not affected by potential confounders such as ‘Fear of crime’, meaning that in regions with more intentional homicides people’s propensity to confide in institutions and strangers will be lower, regardless of individuals’ fear of being victim of a crime.

However, evidence does not support the same conclusion for property crimes. Even though more robberies and vehicle thefts at the NUTS II level have a negative direct effect on social trust (Model 3, p < 0.05), results from Model 4 indicate that confidence in institutions does not mediate the relationship. Indeed, the percentage of the total effect mediated by institutional trust is rather small (around 17%) and insignificant, whereas the direct impact of property crimes remains relevant in the equation (p < 0.01 –even though the size of property crimes’ coefficient strongly decreases). Overall, these findings provide a partial confirmation to H1, as they suggest that only violent crimes are negatively associated with trust towards fellow citizens because of a weaker confidence in the state.

Table 4 presents results from a two-level structural equation model, estimating the intervening role of institutional trust on the relationship between fairness and social trust. In line with the literature, Models 5 and 7 indicate that both impartiality and lack of corruption at the regional level have a strong positive correlation with social trust: regions with lower corruption and more impartiality display higher levels of trust towards fellow citizens at the individual level (p < 0.001), as expected.

**Table 4:** MSEM testing the mediation effect of institutional trust on the relationship between fairness and social trust.

	Model 5 No Mediation	Model 6 With Mediation	Model 7 No Mediation	Model 8 With Mediation
Individual Level				
*SOCIAL TRUST ON*				
Age	0.004* (0.002)	0.002 (0.002)	0.004* (0.002)	0.002 (0.002)
Male	-0.134** (0.043)	-0.102* (0.044)	-0.135** (0.043)	-0.102* (0.044)
Education	0.187*** (0.023)	0.174*** (0.022)	0.188*** (0.023)	0.175*** (0.022)
Unemployment	-0.218*** (0.054)	-0.179*** (0.054)	-0.222*** (0.054)	-0.181*** (0.054)
Perceived Health Status	-0.208*** (0.034)	-0.135*** (0.035)	-0.205*** (0.034)	-0.134*** (0.035)
Social Connections	0.026 (0.022)	0.026 (0.023)	0.026 (0.022)	0.026 (0.023)
Discriminated Group	-0.261** (0.099)	-0.156 (0.095)	-0.266** (0.099)	-0.160 (0.095)
Pro-migrants	0.237*** (0.022)	0.169*** (0.025)	0.236*** (0.022)	0.168*** (0.025)
Fear of Crime	-0.314*** (0.032)	-0.285*** (0.033)	-0.313*** (0.033)	-0.286*** (0.033)
Institutional Trust	-	0.329*** (0.026)	-	0.329*** (0.026)
NUTS II Level				
*INSTITUTIONAL TRUST ON*				
% Migrants from outside EU	-	-10.734** (3.599)	-	-7.452* (3.793)
% Migrants from inside EU	-	-0.656 (5.008)	-	-1.236 (5.020)
% Managers or Professionals	-	-2.323* (1.058)	-	0.088 (0.980)
% Upper degree or above	-	0.008 (0.004)	-	0.008 (0.005)
GDP	-	0.013*** (0.002)	-	0.009*** (0.002)
Population Growth	-	0.002 (0.025)	-	-0.020 (0.022)
Age Median	-	-0.042 (0.032)	-	-0.051 (0.027)
Area in Km2	-	-0.002 (0.002)	-	0.001 (0.002)
Long-term Unemployment	-	-0.008 (0.025)	-	0.045 (0.025)
**Impartiality Pillar**	-	0.304*** **(0.063)**	-	-
**Corruption Pillar**	-	-	-	0.477*** **(0.070)**
*SOCIAL TRUST ON*				
% Migrants from outside EU	-6.292 (3.840)	0.539 (4.627)	-1.859 (3.381)	3.623 (4.253)
% Migrants from inside EU	-1.238 (5.325)	0.138 (5.454)	1.622 (6.001)	3.433 (5.642)
% Managers or Professionals	-0.532 (1.039)	1.061 (0.917)	2.637* (1.067)	2.494** (0.946)
% Upper degree or above	0.006 (0.004)	0.001 (0.003)	0.005 (0.004)	0.001 (0.003)
GDP	0.012*** (0.002)	0.003 (0.003)	0.008** (0.003)	0.001 (0.004)
Population Growth	-0.092** (0.030)	-0.086** (0.027)	-0.132*** (0.032)	-0.111*** (0.030)
Age Median	-0.127*** (0.040)	-0.099** (0.036)	-0.150*** (0.043)	-0.112** (0.041)
Area in Km2	0.001 (0.004)	0.001 (0.004)	0.003 (0.004)	0.003 (0.004)
Long-term Unemployment	-0.001 (0.030)	0.003 (0.025)	0.065 (0.035)	0.025 (0.033)
**Impartiality Pillar**	0.501*** **(0.063)**	0.289*** **(0.063)**	-	-
**Corruption Pillar**	-	-	0.604*** **(0.100)**	**0.234 (0.121)**
**Institutional Trust**	-	0.694***(0.149)	-	0.777*** **(0.198)**
**Impartiality Indirect Effect**	-	0.211*** **(0.065)**	-	-
**% of Total Effect**	-	**42%**	-	-
**Corruption Indirect Effect**	-	-	-	0.371*** **(0.110)**
**% of Total Effect**	-	-	-	**61%**
n individuals (NUTS II)	13,251 (82)	12,840 (82)	13,251 (82)	12,840 (82)
RMSEA	0.030	0.022	0.030	0.022
R2 Between	0.904	0.938	0.877	0.912
R2 Within	0.085	0.143	0.085	0.143

Note. Unstandardized Coefficients. Standard errors in parentheses. MLR.

* *p* ≤ 0.05,

** *p* ≤ 0.01,

*** *p* ≤ 0.001.

Models 6 and 8 account also for mediation of institutional trust at the individual level. Sources: ESS, EUROSTAT, QoG regional data.

In Models 6 and 8, we assess to what extent these correlations are due to institutional trust. Results support H2 and illustrate that the impact of both Corruption and Impartiality are mediated by confidence in institutions, suggesting that widespread unfairness will lead people to trust less each other because of a lower confidence in the institutions (Model 6 and 8). As a matter of fact, Model 8 shows that about 61% of the total effect of the Corruption Pillar on social trust passes through institutional trust. Similar results can be observed for the Impartiality Pillar in Model 6 (about 42% of the total effect is mediated). However, Impartiality maintains a strong and significant direct impact on social trust (p < 0.001) even in the MSEM accounting for the intervening role of institutional trust (Model 6). This indicates that the mediation mechanism works in a weaker way for this dimension of fairness, and that the relationship with social trust might be driven by other factors as well.

Finally, it is important to notice that when we consider other covariates, results point out that the mediation effect could be wider than what assumed in the literature, involving other NUTS II level variables as well. For instance, if we focus on GDP’s impact, it emerges that the variable is strongly mediated: 87% of the total effect is indeed due to the between indirect effect, which is always very significant (p < 0.001) across all equations. Therefore, future research should consider a wider intervening effect of institutional trust, investigating whether it applies to goods unrelated to the ability of the state to act as an effective and fair enforcer.

## Discussion

Following the institutional theoretical framework, this paper provides a first empirical analysis of the intervening role of institutional trust on the association between law-breaking, fairness, and individuals’ propensity to rely on strangers. More specifically, we tested if and to what extent the reported correlations between social trust and impartiality, corruption, violent and property crimes are mediated by institutional trust, using MSEM and data from the European Social Survey (2010), EUROSTAT, and the Quality of Government EU regional data. The article explored fundamental implications of the institution-led perspective and addressed the lack of empirical research on the mediating function of institutional trust.

Results showed a strong intervening role of institutional trust on the relationship between intentional homicides, fairness and social trust, clearly supporting the institutional perspective. However, we also identified some important limitations to this mechanism. In the first place, we found no evidence of mediation of institutional trust concerning property crimes. This indicates that law-breaking correlates with our willingness to rely on others in different ways depending on the type of crime considered. We are likely to distrust institutions when homicide rates are high, as we are less prone to believe that the state can intervene as an effective third-party enforcer. However, the same pattern does not apply to property crimes: frequent robberies or thefts are not associated with a lower confidence in institutions, but only with a lower trust in fellow citizens. Secondly, we observed that the mediation mechanism works more effectively for certain dimensions of fairness: while the association between corruption and social trust was mostly passing through institutional trust, the impartiality pillar was only moderately mediated, and it maintained a significant direct effect on social trust. Finally, evidence indicated that even factors unrelated to safety or fairness issues, such as GDP, might be mediated by institutional trust. In this sense, our study suggests that that the scope of the mediation effect of institutional trust could differ from what originally postulated in the literature, encouraging further research on the topic.

However, our study cannot make any definitive conclusion on the direction of causality, especially as concerns the relationship between institutional and social trust (even though recent empirical evidence supports a causal impact of institutional trust on social trust rather than the other way around [32]). In fact, the statistical techniques currently available for multilevel mediation cannot exclude that the correlational relationships observed are a product of reverse causality (e.g. 2-level non-recursive models do not easily converge when estimating multilevel mediation, while sensitivity analysis for multilevel mediation to assess robustness of predictors has not been developed yet [41, 42]).

Furthermore, we could not employ panel data. This line of analysis was practically unfeasible to test the mediation argument presented, as it would imply the use of a 3-level mediation MSEM (i.e. observations at each point in time would be clustered within individuals, who would be clustered within NUTS II regions), whose complex implementation is still under discussion in the literature [43]. Indeed, while traditional mediation multilevel modeling (MLM) estimates biased coefficients that conflate across levels, MSEM can correctly estimate multilevel mediation. However, in the 3-level mediation case MSEM presents several limitations, and it requires further research to be correctly implemented. For instance, while Preacher and colleagues’ contributions “offer some guidance on choosing appropriate sample sizes in two-level mediation models, […] little is known about the minimum necessary sample size at each level in two- and three-level MSEM” [43]. In addition, “no SEM software is yet […] optimized to fit three-level [mediation] models” [43]. Finally, the paucity of longitudinal datasets gathering information on relevant macro-institutional factors and different forms of trust further indicates how impractical this line of research currently is. In this respect, two main issues limited our choice: (1) the lack of a satisfactory number of waves and cases both at the individual and aggregate levels; (2) the lack of significant variation in values of macro factors (e.g. regional homicide rates) for countries where adequate longitudinal surveys where available (e.g. Switzerland or Denmark). Thus, MSEM mediation analysis was here applied as an exploratory tool rather than a confirmatory causal analysis [44].

Overall, our findings support the correlational validity of the mediation argument, refine the current institutional account of social trust, and offer new directions of research. Future studies should attempt to disentangle the causality issues and better address the mediation effect once appropriate techniques will be fully developed (i.e. 3-level MSEM with mediators at the first and second level [43]).

## Supporting information

S1 File Table A. CFA for institutional trust. Table B. CFA for pro-migrants attitudes. Table C. Descriptive statistics. Table D. MSEM with latent centering assessing mediation of institutional trust on the relationship between violent crimes and social trust. Table E. MSEM with latent centering assessing mediation of institutional trust on the relationship between property crimes and social trust. Table F. MSEM with latent centering assessing mediation of institutional trust on the relationship between impartiality and social trust. Table G. MSEM with latent centering assessing mediation of institutional trust on the relationship between corruption and social trust.
(DOCX)
