# Supplementary material for: Law-breaking, fairness, and generalized trust: The mediating role of trust in institutions
Source: PLoS One. 2019 Aug 7;14(8):e0220160. doi: 10.1371/journal.pone.0220160 (PMC6685600; doi:10.1371/journal.pone.0220160)
Supplement: S1 File — Table A. CFA for institutional trust. Table B. CFA for pro-migrants attitudes. Table C. Descriptive statistics. Table D. MSEM with latent centering assessing mediation of institutional trust on the relationship between violent crimes and social trust. Table E. MSEM with latent centering assessing mediation of institutional trust on the relationship between property crimes and social trust. Table F. MSEM with latent centering assessing mediation of institutional trust on the relationship between impartiality and social trust. Table G. MSEM with latent centering assessing mediation of institutional trust on the relationship between corruption and social trust. (DOCX) [file pone.0220160.s001.docx]

**Supporting Information (S1)**

*Confirmatory Factor Analysis (CFA) of latent variables and descriptive statistics*

In tables A and B, we show factor loadings of variables employed to construct institutional trust and pro-migrants attitudes. Notice that we separate the within and between components of institutional trust in line with Preacher and colleagues [1]. Table C displays the descriptive statistics.

**Table A. CFA for institutional trust.**

|  | Factor Loadings | S.E. | Est./S.E. | Two-tailed p-value |
| --- | --- | --- | --- | --- |
| *Individual level* |  |  |  |  |
| INSTITUTIONAL TRUST BY |  |  |  |  |
| Trust in parties | 1.000 | - | - | - |
| Trust in legal system | 1.012 | 0.024 | 43.070 | 0.000 |
| Trust in Parliament | 1.264 | 0.021 | 61.341 | 0.000 |
| *NUTS II level* |  |  |  |  |
| INSTITUTIONAL TRUST BY |  |  |  |  |
| Trust in parties | 1.000 | - | - | - |
| Trust in legal system | 1.091 | 0.058 | 18.754 | 0.000 |
| Trust in Parliament | 1.035 | 0.044 | 23.387 | 0.000 |

n individuals = 22,023; n NUTS II regions = 122.

**Table B. CFA for pro-migrants attitudes**

|  | Factor Loadings | S.E. | Est./S.E. | Two-tailed p-value |
| --- | --- | --- | --- | --- |
| PRO-MIGRANTS BY |  |  |  |  |
| Migrants good for the economy | 1.000 | - | - | - |
| Country’s cultural life enriched by migrants | 1.066 | 0.022 | 48.548 | 0.000 |
| Migrants make country better place to live | 0.971 | 0.023 | 42.119 | 0.000 |

n individuals = 20,707; n NUTS II regions = 122.

**Table C. Descriptive statistics.**

|  | Mean | S.D. | n individuals (NUTS II) |
| --- | --- | --- | --- |
| Institutional trust (individual level) | 0.00 | 1.58 | 22,023 (122) |
| Institutional trust (NUTS II level) | 0.00 | 0.99 | 22,023 (122) |
| Trust in Parties | 3.18 | 2.35 | 22,602 (122) |
| Trust in the Legal System | 4.47 | 2.65 | 22,461 (122) |
| Trust in the Parliament | 3.95 | 2.54 | 22,505 (122) |
| Pro-migrants | 0.00 | 1.85 | 20,707 (122) |
| Migrants good for the economy | 4.73 | 2.35 | 21,864 (122) |
| Country’s cultural life enriched by migrants | 5.53 | 2.40 | 21,818 (122) |
| Migrants make country better place to live | 4.91 | 2.20 | 21,649 (122) |

*Robustness check*

Asparouhov and Muthén have recently shown that latent centering is important to address bias in multilevel mediation [2, 3]. Thus, we re-run our analyses using latent centering to check the robustness of our findings (see tables D-G). As it can be observed, results are strongly consistent with what presented in tables 3 and 4 in the main article, supporting the same conclusions. Notice that latent centering in Mplus 8.3 relies on Bayesian estimation.

**Table D. MSEM with latent centering assessing mediation of institutional trust on the relationship between violent crimes and social trust.**

|  | Model S1 – no Mediation | | | | | | | | | Model S2 – with Mediation | | | | | | |
| --- | --- | --- | --- | --- | --- | --- | --- | --- | --- | --- | --- | --- | --- | --- | --- | --- |
|  | Est. | | Post S.D. | | Lower 2.5% | | Upper 2.5% | | Est. | | | Post S.D. | | Lower 2.5% | | Upper 2.5% |
| Individual Level |  | |  | |  | |  | |  | | |  | |  | |  |
| *SOCIAL TRUST ON* | | | | | | | | | | | | | | | | |
| Social Connections | 0.033* | | 0.012 | | 0.009 | | 0.057 | | 0.037* | | | 0.011 | | 0.015 | | 0.061 |
| Discriminated Group | -0.284* | | 0.074 | | -0.428 | | -0.136 | | -0.162* | | | 0.072 | | -0.306 | | -0.014 |
| Age | 0.005* | | 0.001 | | 0.002 | | 0.007 | | 0.002* | | | 0.001 | | 0.000 | | 0.004 |
| Male | -0.092* | | 0.035 | | -0.163 | | -0.025 | | -0.064 | | | 0.035 | | -0.128 | | 0.008 |
| Education | 0.210* | | 0.018 | | 0.177 | | 0.245 | | 0.190* | | | 0.017 | | 0.157 | | 0.224 |
| Unemployment | -0.179* | | 0.061 | | -0.302 | | -0.060 | | -0.135* | | | 0.060 | | -0.251 | | -0.014 |
| Fear of Crime | -0.305* | | 0.025 | | -0.352 | | -0.256 | | -0.270* | | | 0.024 | | -0.315 | | -0.220 |
| Perceived Health Status | -0.228* | | 0.022 | | -0.271 | | -0.186 | | -0.166* | | | 0.021 | | -0.207 | | -0.124 |
| Pro-migrants | 0.253* | | 0.011 | | 0.232 | | 0.273 | | 0.178* | | | 0.011 | | 0.156 | | 0.198 |
| Institutional Trust | - | | - | | - | | - | | 0.332* | | | 0.012 | | 0.307 | | 0.356 |
| NUTS II Level |  | |  | |  | |  | |  | | |  | |  | |  |
| *INSTITUTIONAL TRUST ON* | | | | | | | | | | | | | | | | |
| % Upper degree or above | - | | - | | - | | - | | 0.009 | | | 0.005 | | -0.001 | | 0.019 |
| Area in Km2 | - | | - | | - | | - | | 0.000 | | | 0.003 | | -0.005 | | 0.005 |
| Population Growth | - | | - | | - | | - | | -0.156* | | | 0.035 | | -0.227 | | -0.086 |
| Age Median | - | | - | | - | | - | | -0.151* | | | 0.043 | | -0.238 | | -0.065 |
| % Migrants from outside EU | - | | - | | - | | - | | -7.343 | | | 3.983 | | -14.815 | | 0.765 |
| % Migrants from inside EU | - | | - | | - | | - | | 5.376 | | | 5.660 | | -5.698 | | 16.514 |
| % Managers or Professionals | - | | - | | - | | - | | -3.105* | | | 1.242 | | -5.537 | | -0.695 |
| GDP | - | | - | | - | | - | | 0.021* | | | 0.002 | | 0.017 | | 0.025 |
| Long-term Unemployment | - | | - | | - | | - | | -0.003 | | | 0.033 | | -0.066 | | 0.063 |
| **Intentional Homicides** | **-** | | **-** | | **-** | | **-** | | **-0.358*** | | | **0.109** | | **-0.575** | | **-0.159** |
| *SOCIAL TRUST ON* | | | | | | | | | | | | | | | | |
| % Upper degree or above | 0.005 | | 0.005 | | -0.006 | | 0.015 | | -0.003 | | | 0.004 | | -0.011 | | 0.005 |
| Area in Km2 | 0.003 | | 0.003 | | -0.003 | | 0.008 | | 0.003 | | | 0.002 | | -0.002 | | 0.007 |
| Population Growth | -0.191* | | 0.039 | | -0.270 | | -0.120 | | -0.039 | | | 0.033 | | -0.107 | | 0.024 |
| Age Median | -0.182* | | 0.049 | | -0.278 | | -0.085 | | -0.040 | | | 0.039 | | -0.118 | | 0.037 |
| % Migrants from outside EU | 0.563 | | 4.361 | | -8.323 | | 8.650 | | 6.842 | | | 3.448 | | -0.077 | | 13.638 |
| % Migrants from inside EU | 7.110 | | 6.272 | | -4.466 | | 19.934 | | 2.426 | | | 4.820 | | -7.003 | | 12.045 |
| % Managers or Professionals | -0.619 | | 1.420 | | -3.361 | | 2.190 | | 2.201* | | | 1.078 | | 0.148 | | 4.345 |
| GDP | 0.016* | | 0.002 | | 0.011 | | 0.020 | | -0.004 | | | 0.003 | | -0.009 | | 0.002 |
| Long-term Unemployment | -0.030 | | 0.037 | | -0.104 | | 0.040 | | -0.024 | | | 0.027 | | -0.080 | | 0.029 |
| **Intentional Homicides** | **-0.379*** | | **0.121** | | **-0.617** | | **-0.141** | | **-0.043** | | | **0.098** | | **-0.233** | | **0.151** |
| **Institutional Trust** | **-** | | **-** | | **-** | | **-** | | **0.935*** | | | **0.119** | | **0.699** | | **1.167** |
| **Intentional Homicides Indirect Effect** | **-** | | **-** | | **-** | | **-** | | **-0.333*** | | | **0.109** | | **-0.567** | | **-0.138** |
| **% of Total Effect** | **-** | | **-** | | **-** | | **-** | | **89%** | | | | |  | |  |
| N individuals (NUTS II) | 17,087 (99) | | | | | | | | 16,534 (99) | | | | | | | |
|  |  |  | |  | |  | |  | | |  | |  | |  | |

Note. Unstandardized Coefficients. Bayes Estimator. * indicates a non-null (or significant) effect; 95% C.I. Model S2 accounts also for mediation of institutional trust at the individual level. Sources: ESS, EUROSTAT, QoG regional data.

**Table E. MSEM with latent centering assessing mediation of institutional trust on the relationship between property crimes and social trust.**

|  | Model S3 – no Mediation | | | | | | | | | Model S4 – with Mediation | | | | | | |
| --- | --- | --- | --- | --- | --- | --- | --- | --- | --- | --- | --- | --- | --- | --- | --- | --- |
|  | Est. | | Post S.D. | | Lower 2.5% | | Upper 2.5% | | Est. | | | Post S.D. | | Lower 2.5% | | Upper 2.5% |
| Individual Level |  | |  | |  | |  | |  | | |  | |  | |  |
| *SOCIAL TRUST ON* | | | | | | | | | | | | | | | | |
| Social Connections | 0.035* | | 0.011 | | 0.013 | | 0.057 | | 0.034* | | | 0.011 | | 0.012 | | 0.056 |
| Discriminated Group | -0.294* | | 0.069 | | -0.429 | | -0.159 | | -0.171* | | | 0.068 | | -0.305 | | -0.039 |
| Age | 0.005* | | 0.001 | | 0.002 | | 0.007 | | 0.002* | | | 0.001 | | 0.0001 | | 0.004 |
| Male | -0.096* | | 0.032 | | -0.160 | | -0.034 | | -0.078* | | | 0.032 | | -0.140 | | -0.015 |
| Education | 0.209* | | 0.016 | | 0.177 | | 0.241 | | 0.177* | | | 0.016 | | 0.147 | | 0.208 |
| Unemployment | -0.181* | | 0.058 | | -0.297 | | -0.071 | | -0.139* | | | 0.058 | | -0.251 | | -0.023 |
| Fear of Crime | -0.309* | | 0.023 | | -0.352 | | -0.264 | | -0.278* | | | 0.023 | | -0.323 | | -0.233 |
| Perceived Health Status | -0.223* | | 0.020 | | -0.263 | | -0.185 | | -0.167* | | | 0.020 | | -0.207 | | -0.129 |
| Pro-migrants | 0.258* | | 0.010 | | 0.238 | | 0.278 | | 0.179* | | | 0.011 | | 0.158 | | 0.200 |
| Institutional Trust | - | | - | | - | | - | | 0.339* | | | 0.012 | | 0.316 | | 0.363 |
| NUTS II Level |  | |  | |  | |  | |  | | |  | |  | |  |
| *INSTITUTIONAL TRUST ON* | | | | | | | | | | | | | | | | |
| % Upper degree or above | - | | - | | - | | - | | 0.005 | | | 0.006 | | -0.006 | | 0.016 |
| Area in Km2 | - | | - | | - | | - | | 0.000 | | | 0.003 | | -0.006 | | 0.006 |
| Population Growth | - | | - | | - | | - | | -0.102* | | | 0.040 | | -0.183 | | -0.026 |
| Age Median | - | | - | | - | | - | | -0.131* | | | 0.050 | | -0.232 | | -0.034 |
| % Migrants from outside EU | - | | - | | - | | - | | -11.86* | | | 4.453 | | -20.908 | | -3.384 |
| % Migrants from inside EU | - | | - | | - | | - | | 10.063 | | | 6.698 | | -3.258 | | 23.388 |
| % Managers or Professionals | - | | - | | - | | - | | -3.459* | | | 1.415 | | -6.212 | | -0.716 |
| GDP | - | | - | | - | | - | | 0.022* | | | 0.003 | | 0.016 | | 0.027 |
| Long-term Unemployment | - | | - | | - | | - | | -0.035 | | | 0.040 | | -0.113 | | 0.043 |
| **Property Crimes** | **-** | | **-** | | **-** | | **-** | | **0.000** | | | **0.000** | | **-0.001** | | **0.001** |
| *SOCIAL TRUST ON* | | | | | | | | | | | | | | | | |
| % Upper degree or above | -0.001 | | 0.005 | | -0.012 | | 0.009 | | -0.005 | | | 0.004 | | -0.012 | | 0.002 |
| Area in Km2 | 0.001 | | 0.003 | | -0.004 | | 0.007 | | 0.001 | | | 0.002 | | -0.002 | | 0.005 |
| Population Growth | -0.126* | | 0.039 | | -0.205 | | -0.053 | | -0.025 | | | 0.028 | | -0.080 | | 0.03 |
| Age Median | -0.149* | | 0.049 | | -0.246 | | -0.055 | | -0.025 | | | 0.036 | | -0.094 | | 0.046 |
| % Migrants from outside EU | -3.673 | | 4.240 | | -12.164 | | 4.747 | | 6.532* | | | 3.194 | | 0.098 | | 12.703 |
| % Migrants from inside EU | 10.504 | | 6.352 | | -1.898 | | 23.772 | | 1.571 | | | 4.750 | | -7.656 | | 10.8 |
| % Managers or Professionals | -0.227 | | 1.385 | | -2.832 | | 2.564 | | 2.793* | | | 0.995 | | 0.849 | | 4.766 |
| GDP | 0.017* | | 0.002 | | 0.012 | | 0.022 | | -0.002 | | | 0.003 | | -0.007 | | 0.003 |
| Long-term Unemployment | -0.051 | | 0.038 | | -0.124 | | 0.019 | | -0.017 | | | 0.026 | | -0.069 | | 0.035 |
| **Property Crimes** | **-0.001*** | | **0.000** | | **-0.002** | | **-0.0001** | | **-0.001*** | | | **0.000** | | **-0.001** | | **-0.0001** |
| **Institutional Trust** | **-** | | **-** | | **-** | | **-** | | **0.876*** | | | **0.094** | | **0.699** | | **1.067** |
| **Property Crimes Indirect Effect** | **-** | | **-** | | **-** | | **-** | | **-0.0002** | | | **0.0004** | | **-0.0009** | | **0.0006** |
| **% of Total Effect** | **-** | | **-** | | **-** | | **-** | | **17%** | | | | |  | |  |
| N individuals (NUTS II) | 18,734 (110) | | | | | | | | 18,148 (110) | | | | | | | |
|  |  |  | |  | |  | |  | | |  | |  | |  | |

Note. Unstandardized Coefficients. Bayes Estimator. * indicates a non-null (or significant) effect; 95% C.I. Model S4 accounts also for mediation of institutional trust at the individual level. Sources: ESS, EUROSTAT, QoG regional data.

**Table F. MSEM with latent centering assessing mediation of institutional trust on the relationship between impartiality and social trust.**

|  | Model S5 – no Mediation | | | | | | | | | Model S6 – with Mediation | | | | | | |
| --- | --- | --- | --- | --- | --- | --- | --- | --- | --- | --- | --- | --- | --- | --- | --- | --- |
|  | Est. | | Post S.D. | | Lower 2.5% | | Upper 2.5% | | Est. | | | Post S.D. | | Lower 2.5% | | Upper 2.5% |
| Individual Level |  | |  | |  | |  | |  | | |  | |  | |  |
| *SOCIAL TRUST ON* | | | | | | | | | | | | | | | | |
| Social Connections | 0.029* | | 0.013 | | 0.004 | | 0.055 | | 0.028* | | | 0.013 | | 0.002 | | 0.054 |
| Discriminated Group | -0.250* | | 0.086 | | -0.422 | | -0.081 | | -0.148 | | | 0.083 | | -0.312 | | 0.012 |
| Age | 0.004* | | 0.001 | | 0.001 | | 0.007 | | 0.002 | | | 0.001 | | -0.001 | | 0.004 |
| Male | -0.138* | | 0.039 | | -0.212 | | -0.062 | | -0.104* | | | 0.039 | | -0.181 | | -0.027 |
| Education | 0.200* | | 0.019 | | 0.161 | | 0.237 | | 0.183* | | | 0.019 | | 0.146 | | 0.221 |
| Unemployment | -0.216* | | 0.068 | | -0.351 | | -0.078 | | -0.179* | | | 0.067 | | -0.310 | | -0.047 |
| Fear of Crime | -0.326* | | 0.026 | | -0.379 | | -0.276 | | -0.295* | | | 0.027 | | -0.348 | | -0.243 |
| Perceived Health Status | -0.200* | | 0.024 | | -0.245 | | -0.154 | | -0.139* | | | 0.024 | | -0.187 | | -0.092 |
| Pro-migrants | 0.234* | | 0.011 | | 0.211 | | 0.257 | | 0.165* | | | 0.012 | | 0.141 | | 0.190 |
| Institutional Trust | - | | - | | - | | - | | 0.329* | | | 0.014 | | 0.302 | | 0.356 |
| NUTS II Level |  | |  | |  | |  | |  | | |  | |  | |  |
| *INSTITUTIONAL TRUST ON* | | | | | | | | | | | | | | | | |
| % Upper degree or above | - | | - | | - | | - | | 0.008* | | | 0.004 | | 0.0001 | | 0.017 |
| Area in Km2 | - | | - | | - | | - | | -0.002 | | | 0.003 | | -0.007 | | 0.004 |
| Population Growth | - | | - | | - | | - | | 0.000 | | | 0.037 | | -0.073 | | 0.073 |
| Age Median | - | | - | | - | | - | | -0.042 | | | 0.043 | | -0.127 | | 0.042 |
| % Migrants from outside EU | - | | - | | - | | - | | -10.93* | | | 3.618 | | -18.189 | | -4.004 |
| % Migrants from inside EU | - | | - | | - | | - | | -0.930 | | | 5.343 | | -11.267 | | 9.746 |
| % Managers or Professionals | - | | - | | - | | - | | -2.387* | | | 1.068 | | -4.497 | | -0.300 |
| GDP | - | | - | | - | | - | | 0.014* | | | 0.002 | | 0.009 | | 0.018 |
| Long-term Unemployment | - | | - | | - | | - | | -0.001 | | | 0.027 | | -0.055 | | 0.053 |
| **Impartiality** | **-** | | **-** | | **-** | | **-** | | **0.310*** | | | **0.057** | | **0.201** | | **0.425** |
| *SOCIAL TRUST ON* | | | | | | | | | | | | | | | | |
| % Upper degree or above | 0.006 | | 0.005 | | -0.004 | | 0.016 | | 0.001 | | | 0.004 | | -0.008 | | 0.009 |
| Area in Km2 | 0.000 | | 0.003 | | -0.007 | | 0.007 | | 0.002 | | | 0.003 | | -0.004 | | 0.008 |
| Population Growth | -0.095* | | 0.041 | | -0.174 | | -0.014 | | -0.085* | | | 0.036 | | -0.156 | | -0.014 |
| Age Median | -0.130* | | 0.049 | | -0.227 | | -0.036 | | -0.099* | | | 0.043 | | -0.184 | | -0.015 |
| % Migrants from outside EU | -6.284 | | 4.030 | | -14.536 | | 1.605 | | 0.620 | | | 3.939 | | -7.135 | | 8.377 |
| % Migrants from inside EU | -0.909 | | 6.265 | | -12.978 | | 11.621 | | 0.373 | | | 5.491 | | -10.315 | | 11.168 |
| % Managers or Professionals | -0.517 | | 1.195 | | -2.850 | | 1.739 | | 1.137 | | | 1.105 | | -0.995 | | 3.352 |
| GDP | 0.012* | | 0.002 | | 0.008 | | 0.017 | | 0.003 | | | 0.003 | | -0.003 | | 0.009 |
| Long-term Unemployment | -0.002 | | 0.029 | | -0.060 | | 0.053 | | -0.003 | | | 0.027 | | -0.055 | | 0.049 |
| **Impartiality** | **0.497*** | | **0.062** | | **0.365** | | **0.610** | | **0.281*** | | | **0.076** | | **0.128** | | **0.428** |
| **Institutional Trust** | **-** | | **-** | | **-** | | **-** | | **0.689*** | | | **0.166** | | **0.374** | | **1.027** |
| **Impartiality Indirect Effect** | **-** | | **-** | | **-** | | **-** | | **0.210*** | | | **0.064** | | **0.103** | | **0.353** |
| **% of Total Effect** | **-** | | **-** | | **-** | | **-** | | **43%** | | | | |  | |  |
| N individuals (NUTS II) | 13,251 (82) | | | | | | | | 12,840 (82) | | | | | | | |
|  |  |  | |  | |  | |  | | |  | |  | |  | |

Note. Unstandardized Coefficients. Bayes Estimator. * indicates a non-null (or significant) effect; 95% C.I. Model S6 accounts also for mediation of institutional trust at the individual level. Sources: ESS, EUROSTAT, QoG regional data.

**Table G. MSEM with latent centering assessing mediation of institutional trust on the relationship between corruption and social trust.**

|  | Model S7 – no Mediation | | | | | | | | | Model S8 – with Mediation | | | | | | |
| --- | --- | --- | --- | --- | --- | --- | --- | --- | --- | --- | --- | --- | --- | --- | --- | --- |
|  | Est. | | Post S.D. | | Lower 2.5% | | Upper 2.5% | | Est. | | | Post S.D. | | Lower 2.5% | | Upper 2.5% |
| Individual Level |  | |  | |  | |  | |  | | |  | |  | |  |
| *SOCIAL TRUST ON* | | | | | | | | | | | | | | | | |
| Social Connections | 0.030* | | 0.013 | | 0.005 | | 0.055 | | 0.029* | | | 0.013 | | 0.004 | | 0.056 |
| Discriminated Group | -0.254* | | 0.086 | | -0.426 | | -0.086 | | -0.152 | | | 0.083 | | -0.313 | | 0.011 |
| Age | 0.004* | | 0.001 | | 0.001 | | 0.006 | | 0.002 | | | 0.001 | | -0.001 | | 0.004 |
| Male | -0.139* | | 0.039 | | -0.212 | | -0.063 | | -0.103* | | | 0.039 | | -0.178 | | -0.025 |
| Education | 0.200* | | 0.019 | | 0.162 | | 0.238 | | 0.184* | | | 0.019 | | 0.146 | | 0.221 |
| Unemployment | -0.219* | | 0.068 | | -0.353 | | -0.082 | | -0.176* | | | 0.067 | | -0.314 | | -0.045 |
| Fear of Crime | -0.325* | | 0.026 | | -0.378 | | -0.275 | | -0.294* | | | 0.027 | | -0.346 | | -0.241 |
| Perceived Health Status | -0.198* | | 0.024 | | -0.243 | | -0.152 | | -0.137* | | | 0.024 | | -0.184 | | -0.089 |
| Pro-migrants | 0.233* | | 0.012 | | 0.210 | | 0.255 | | 0.165* | | | 0.013 | | 0.140 | | 0.190 |
| Institutional Trust | - | | - | | - | | - | | 0.329* | | | 0.014 | | 0.302 | | 0.357 |
| NUTS II Level |  | |  | |  | |  | |  | | |  | |  | |  |
| *INSTITUTIONAL TRUST ON* | | | | | | | | | | | | | | | | |
| % Upper degree or above | - | | - | | - | | - | | 0.009* | | | 0.004 | | 0.001 | | 0.017 |
| Area in Km2 | - | | - | | - | | - | | 0.000 | | | 0.003 | | -0.005 | | 0.005 |
| Population Growth | - | | - | | - | | - | | -0.025 | | | 0.033 | | -0.09 | | 0.039 |
| Age Median | - | | - | | - | | - | | -0.052 | | | 0.039 | | -0.128 | | 0.024 |
| % Migrants from outside EU | - | | - | | - | | - | | -7.415* | | | 3.312 | | -14.085 | | -0.920 |
| % Migrants from inside EU | - | | - | | - | | - | | -1.679 | | | 4.955 | | -11.679 | | 7.797 |
| % Managers or Professionals | - | | - | | - | | - | | -0.003 | | | 1.024 | | -2.007 | | 2.018 |
| GDP | - | | - | | - | | - | | 0.009* | | | 0.002 | | 0.006 | | 0.014 |
| Long-term Unemployment | - | | - | | - | | - | | 0.057* | | | 0.027 | | 0.003 | | 0.114 |
| **Corruption** | **-** | | **-** | | **-** | | **-** | | **0.490*** | | | **0.073** | | **0.349** | | **0.633** |
| *SOCIAL TRUST ON* | | | | | | | | | | | | | | | | |
| % Upper degree or above | 0.006 | | 0.005 | | -0.005 | | 0.016 | | -0.001 | | | 0.005 | | -0.010 | | 0.008 |
| Area in Km2 | 0.003 | | 0.004 | | -0.004 | | 0.010 | | 0.003 | | | 0.003 | | -0.003 | | 0.010 |
| Population Growth | -0.136* | | 0.044 | | -0.219 | | -0.048 | | -0.107* | | | 0.040 | | -0.186 | | -0.029 |
| Age Median | -0.153* | | 0.052 | | -0.253 | | -0.052 | | -0.109* | | | 0.048 | | -0.203 | | -0.015 |
| % Migrants from outside EU | -1.631 | | 4.353 | | -10.252 | | 6.949 | | 3.902 | | | 4.225 | | -4.174 | | 12.332 |
| % Migrants from inside EU | 1.541 | | 6.679 | | -11.431 | | 14.705 | | 3.603 | | | 5.976 | | -8.293 | | 15.284 |
| % Managers or Professionals | 2.567 | | 1.361 | | -0.145 | | 5.253 | | 2.551* | | | 1.211 | | 0.179 | | 4.918 |
| GDP | 0.008* | | 0.003 | | 0.003 | | 0.013 | | 0.000 | | | 0.003 | | -0.006 | | 0.006 |
| Long-term Unemployment | 0.064 | | 0.035 | | -0.009 | | 0.128 | | 0.014 | | | 0.034 | | -0.054 | | 0.082 |
| **Corruption** | **0.601*** | | **0.089** | | **0.419** | | **0.766** | | **0.211** | | | **0.125** | | **-0.043** | | **0.453** |
| **Institutional Trust** | **-** | | **-** | | **-** | | **-** | | **0.797*** | | | **0.207** | | **0.408** | | **1.222** |
| **Corruption Indirect Effect** | **-** | | **-** | | **-** | | **-** | | **0.387*** | | | **0.112** | | **0.193** | | **0.635** |
| **% of Total Effect** | **-** | | **-** | | **-** | | **-** | | **65%** | | | | |  | |  |
| N individuals (NUTS II) | 13,251 (82) | | | | | | | | 12,840 (82) | | | | | | | |
|  |  |  | |  | |  | |  | | |  | |  | |  | |

Note. Unstandardized Coefficients. Bayes Estimator. * indicates a non-null (or significant) effect; 95% C.I. Model S8 accounts also for mediation of institutional trust at the individual level. Sources: ESS, EUROSTAT, QoG regional data.

**Reference List**

1. Preacher KJ, Zyphur MJ, Zhang Z. A general multilevel SEM framework for assessing multilevel mediation. Psychological methods. 2010;15(3):209.

2. Asparouhov T, Muthén B. Latent variable centering of predictors and mediators in multilevel and time-series models. Structural Equation Modeling: A Multidisciplinary Journal. 2019;26(1):119-42.

3. Lüdtke O, Marsh HW, Robitzsch A, Trautwein U, Asparouhov T, Muthén B. The multilevel latent covariate model: A new, more reliable approach to group-level effects in contextual studies. Psychological methods. 2008;13(3):203.
